# Event-related potentials reveal rapid registration of features of infrequent changes during change blindness

**DOI:** 10.1186/1744-9081-6-12

**Published:** 2010-02-09

**Authors:** Pessi Lyyra, Jan Wikgren, Piia Astikainen

**Affiliations:** 1 Department of Psychology, PO Box 35, FI-40014 University of Jyväskylä, Jyväskylä, Finland

## Abstract

**Background:**

Change blindness refers to a failure to detect changes between consecutively presented images separated by, for example, a brief blank screen. As an explanation of change blindness, it has been suggested that our representations of the environment are sparse outside focal attention and even that changed features may not be represented at all. In order to find electrophysiological evidence of neural representations of changed features during change blindness, we recorded event-related potentials (ERPs) in adults in an oddball variant of the change blindness flicker paradigm.

**Methods:**

ERPs were recorded when subjects performed a change detection task in which the modified images were infrequently interspersed (p = .2) among the frequently (p = .8) presented unmodified images. Responses to modified and unmodified images were compared in the time window of 60-100 ms after stimulus onset.

**Results:**

ERPs to infrequent modified images were found to differ in amplitude from those to frequent unmodified images at the midline electrodes (Fz, Pz, Cz and Oz) at the latency of 60-100 ms even when subjects were unaware of changes (change blindness).

**Conclusions:**

The results suggest that the brain registers changes very rapidly, and that changed features in images are neurally represented even without participants' ability to report them.

## Background

Experimental psychologists have recently demonstrated a noteworthy failure to detect changes in visual environment, named "change blindness" [1,2]. The best known method of experimentally inducing change blindness is the flicker paradigm [3], in which a briefly presented blank screen separates presentations of original and modified images.

The phenomenon of change blindness has led some researchers to theorize that we can only have detailed visual representations of our environment inside the focus of attention and in change blindness the unnoticed changed features would not be represented at all [1,4]. Call these no-representation accounts of change blindness. In other words, explicit change detection would only be possible when top-down focal attention is directed to the locus of change [3]. Consequently, this account predicts that the changes are not registered even implicitly, as this would only be possible by having some representation of the changed features. However, some experimental evidence from gaze-tracking [5] and forced-choice tasks [6-8] has pointed towards the possibility that some implicit bottom-up processes may guide visual perception even during change blindness.

Change blindness has also attracted the interest of neuroscientists [7,9-20]. Investigating brain responses could be even more informative than behavioral measures about the causes of change blindness. Indeed, any brain response elicited by changed features during change blindness would count as counter-evidence to the no-representation account [1,4]. Some researchers have reported observing differential brain activity for changes during change blindness compared to no-change condition [7,9,10,12-16]. For example, evidence from event-related potentials (ERPs) of implicit change detection was provided in a study by Fernandez-Duque et al. [7], in which a continuous flicker paradigm was used. The authors compared responses at the latency of 240-300 ms in two separate stimulus blocks: one with no changes and the other in which unnoticed changes were present. However, responses to modified and unmodified images were not compared to each other, but instead unmodified pictures in these two conditions. For this reason it is possible that the result reflected implicit processing of the presence of changes, but not directly implicit responses to changed features in stimuli. In another ERP-study, Eimer & Mazza ([10], see also [13]) investigated brain responses to noticed and unnoticed changes using the S1-S2, or "one-shot", flicker paradigm in which the changes occur in S2. They also compared responses to S2 that contained unnoticed changes (change blindness) with responses to S2 that did not contain changes, when participants so correctly reported. The authors observed differences in responses at the early latencies of 30-80 and 90-130 ms after stimulus onset, possibly evoked by unnoticed changed features in the stimuli. However, in their setup, they could not exclude the possibility that effects of task preparation were responsible for this finding. They suspected that subjects' preparation to the task was systematically worse in change blindness trials than in trials in which participants correctly reported the absence of change.

In order to avoid the above mentioned problem, we employed a novel combination of experimental paradigms aimed to reveal implicit detection of changes during change blindness. An *oddball *version of the continuous flicker paradigm was applied, so that changes were infrequent and pseudo-randomly presented, unlike in the standard version of the S1-S2 flicker paradigm in which the stimulus types are presented pair wise with equal probabilities. The advantage of the oddball paradigm is that it allows comparison of the responses to different stimulus types (modified and unmodified images) which are presented in the same stimulus sequence and in which the occurrences of the changes cannot be predicted.

The oddball paradigm has previously been used in the studies of visual mismatch negativity (vMMN) [21]). However, we did not expect to see any vMMN for three main reasons. First of all, no trace of it was observed in a previous study investigating it in the change blindness condition [11]. Second, vMMN is usually elicited in a condition in which changes are not searched for, and thus not attended, but instead the subjects are concentrating on a primary task. Third, the stimulus material (natural scenes) itself differed from those of typical vMMN studies where the stimuli are usually simpler, for example changes are presented in a color of an object [22] or in an orientation of a bar [23].

The no-representation account of change blindness [4] predicts that the modified and unmodified images elicit ERPs of equal magnitude during change blindness. As mentioned, differential ERPs to changed features before subjects' conscious perception of the changes will be counter-evidence for no-representation theories of change blindness. Therefore, we hypothesized that despite the elimination of possible differences in task preparation, unnoticed changes in visual stimuli would evoke differences in brain responses, as observed, e.g., in the study by Eimer and Mazza [10].

## Materials and methods

### Participants

Fourteen volunteers, eight female and five male with age distribution of 19-33 years (mean age 22.9 years) participated in the study. All of them had normal or corrected-to-normal vision. The data of one participant, who did not follow the task instructions, were discarded. A written informed consent was obtained from the participants before the experimental treatment. The study conforms to The Code of Ethics of the World Medical Association (Declaration of Helsinki).

### Procedure

**Figure 1:**
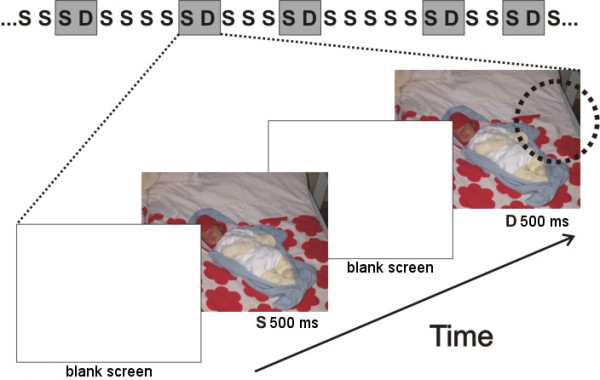
**Illustration of the stimulus paradigm applied**. This is an example of the images applied in the experiment. The succession of the stimuli is depicted uppermost with gray rectangles representing stimuli during which the EEG data were recorded. The duration of the standard (S) and deviant (D) images is 500 ms and that of the blank screen 100 ms. The dotted circle indicates the site of the modification in the example of a deviant image.

During recordings, the participants were seated in a chair in a dimly lit room. They viewed the stimuli on a 17" monitor at a distance of approximately one meter. We chose to use images of size 8° × 11°, representing complex natural scenes (a sample pair of images with indicated change is given in Figure 1), as stimuli to allow as large changes as possible, as it has been shown that the size of targets affects both the amplitude and the latency of the responses to them [24]. Changes consisted in the appearance or disappearance of objects, or in a change of their position or color. All the images were shown to induce change blindness in a pilot study before the actual experiment. Since the changes in images were as large as possible, we used a variety of change types and locations in order to maximize the amount of change blindness trials, as it has been found that occurrences of previous changes can serve as cues for detection of subsequent changes [25]. We aimed to avoid the possible threat that the location or type of change would have any cueing effect by constructing different types of changes in different locations and randomized them across the blocks.

Images were presented for 500 ms, separated by 100-ms non-stimulated interval (stimulus onset asynchrony thus 600 ms). Such a short non-stimulated interval was used to prevent memory decay potentially affecting implicit processing of changed features. In oddball condition, a changed (deviant, p = .2) picture was infrequently interspersed between frequently presented (standard, p = .8) pictures. Standards and deviants were presented pseudo-randomly with the restriction that there were up to seven but no less than three standards between consecutive deviants.

The subjects were familiarized with the task by administration of a rehearsal block similar to the ones used in the actual experiment, and data recorded from the rehearsal blocks were not used in the analyses. The actual experiment consisted of ten stimulus blocks, each block containing 250 stimuli (50 deviants). A break followed each stimulus block and the next block in the series was shown on the participant's request. To prevent effects of novelty for the first standard stimuli, a preparatory series of at least ten standard stimuli were presented before the appearance of the first deviant stimulus. The order of presentation of the ten blocks was randomized across the participants. An illustration of the stimulus condition is given in Figure 1.

The participants were instructed to search for an infrequent change in the pictures and to report identification of the change by pressing a button. They were instructed to press the button only when they identified the change for the first time, subsequent identifications of changes did not require responses. After the initial identification, the participants were instructed to ignore the infrequently occurring identified change and search for more changes to make the attention mode of the change identification condition correspond to the change blindness condition (search changes versus focus on changes [7]).

### EEG-recordings and data-analysis

Electroencephalography (EEG) was recorded on four channels using an elastic cap (Electrocap) with Ag/AgCl electrodes, from the international 10/20 system sites Fz, Cz, Pz and Oz. The linked left and right mastoids served as reference electrodes and one electrode located on the forehead as a ground electrode. The signals were amplified 5,000 times and band-pass-filtered with 0.1 to 30 Hz, and sampled continuously at 500 Hz.

The recorded EEG sweep consisted of a time interval from 100 ms before to 370 ms after stimulus onset for one deviant and the standard immediately preceding it. A 50-ms pre-stimulus period served as the baseline. Thus, in the analyses, there was an equal number of standards and deviants. The standard-deviant pair immediately preceding pressing of the button was considered the moment when the subject consciously noticed the change. This stimulus pair was excluded from the analysis [7]. The preceding trials were considered the change blindness trials, and the trials starting from the button press were considered the change identification trials. Sweeps containing artifacts (maximum difference of values within the sweep exceeding 100 μV in any electrode) were discarded, the average rejection rate being approximately 34%.

**Figure 2:**
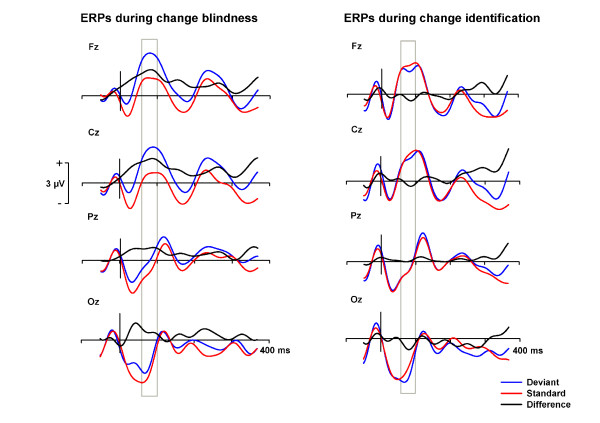
**Grand averaged ERPs to deviants (blue line) and standards (red line) during change blindness and change identification**. The black lines show the differential ERPs (ERPs to deviants minus ERPs to standards). The time window for extracting the mean values for the repeated measures MANOVA is marked with the gray rectangle. The y-axis shows the stimulus onset.

For the statistical analyses, a time window from 60 ms to 100 ms from stimulus onset was determined on the basis of the waveforms of grand-average ERPs (Figure 2) and the study of Eimer & Mazza [7]. Mean amplitude values for standard and deviant ERPs were extracted. The resulting mean values were analyzed by multivariate analyses of variance (MANOVA) for repeated measures with Electrode site (Fz, Cz, Pz and Oz), Stimulus type (standard, deviant) and Awareness (change blindness, change identification) as factors in each window. An alpha level of .05 was used in all the analyses.

In order to complement the deflection analysis with a temporal analysis we used global waveform analysis, an approach that preserves the temporal resolution of EEG (here, sampling frequency of 500 Hz) [18,26]. This approach does not qualify as a reliable identification procedure of ERP effects but it does qualify as a method of reliably revealing the temporal dynamics of the EEG by estimating the onsets and offsets of ERP effects, which are neglected by an inspection applying mean or peak values in an analysis window. We ran point-by-point paired t-tests between responses to standards and deviants in each electrode from the stimulus onset to the end of the measurement window (0-370 ms). To counteract the likelihood of exaggerated significant values associated with multiple t-tests an alpha level of .01 in at least 10 consecutive data points (20 ms) (e.g. [27]) was required to consider modulations in waveforms to be present.

## Results

### Behavioral data

In an image block, the average number of deviant pictures preceding explicit report of change detection (the number of presented changes during change blindness before subjects indicated change detection by a button press) was 14.27 (SEM = 1.29) out of 50. Since data concerning one pre-report presentation of a deviant picture was discounted from the analysis, the mean number of responses to deviants was 13.27 per image type, and thus 133 responses for the change blindness condition, and 357 responses for the change identification condition per subject in average.

### Electrophysiological data

The grand average ERPs to standards and those to deviants are shown in Figure 2 for change blindness and change identification, together with the grand-average difference waves where responses to standards were subtracted from responses to deviants.

A 3-way MANOVA, Awareness (change blindness, change identification) * Stimulus type (standard, deviant) * Electrode site (Fz, Cz, Pz, Oz) at the time window of 60-100 ms from the stimulus onset revealed main effects of Electrode site, *F*(3, 10) = 22.446, *p *< .001, Stimulus type, *F*(1, 12) = 8.887, *p *< .011, and Awareness, *F*(1, 12) = 22.446, *p *< .001. The effect of Electrode site indicated that responses were more positive at the anterior than posterior electrode sites. Also the Stimulus type * Awareness was significant, *F*(1, 12) = 6.638, *p *< .024, suggesting that changes were processed differently during change blindness and change identification.

For change blindness trials (Figure 2), a further analysis with Stimulus type (standard, deviant) revealed a significant main effect of Stimulus type, t(12) = 3.185, *p *= .008, indicating that the presence of changes modulated brain responses despite the inability to report on the changes. The responses were more positive to deviants compared to standards at all the recording sites (mean differences in amplitudes 1.73 μV, averaged across all electrode sites; see also Figure 2).

For the change identification trials (Figure 2), a paired t-test for the Stimulus type (standard, deviant), t(12) = 1.399, *p *= .187, did not reach significance.

On the basis of grand-average and difference ERPs (Figure 2), the observed effect during change blindness seems to linger over the anterior electrodes after the peak at the latency of 60-100 ms as compared to the most posterior electrode site of Oz. To examine temporal features of the differences between responses to standards and deviants in global waveforms, we ran two-tailed paired point-by-point t-tests for the data separately in each electrode. Such epochs in which responses differed for deviants and standards were found for three electrode sites, Fz (70-104 ms, all p < .01), Cz (76-104, all p < .01), and Pz (70-100 ms, all p < .01). At the Oz, the criterion of the alpha level under .01 was not quite reached, but the difference came close to significance at 66-86 ms (all p < .02).

No epoch reached significance in the point-by-point temporal analysis for the change identification trials.

## Discussion

We studied electrophysiological correlates of change blindness in the oddball variant of the flicker paradigm during change blindness, i.e. stimulus presentation during which the subjects did not notice the change, and during which they could not anticipate when the changes would occur. Intriguingly, and compatible with our hypothesis, we found that even during change blindness the occurrences of random changes modulated electrical brain responses at all electrode sites. There was a global positive difference at the latency of less than 100 ms in ERPs to infrequent images containing changes (deviants) compared to frequently presented images without changes (standards), indicating that the changed features are somehow represented in the brain even in the absence of the ability to anticipate or report on the occurrences of the changes. This difference in ERPs was confined to the change blindness situation.

Our results cannot be directly compared to the results of those experiments in which the S1-S2 and traditional continuous flicker of paradigms were applied. This is because search behavior in S1-S2 and traditional continuous flicker paradigms may differ from those in the oddball paradigm. In that sense, changes differ in salience and infrequent changes may require different comparison mechanisms, e.g. searching for a violation in a rule rather than serial comparison of elements, or more sustained attention to specific locations in images. However, the search for implicit representations of changed features during change blindness is not affected, even if search behavior may differ from each other in these different conditions. Any registration of changes still indicates that the changed features are represented at some level in the brain. Infrequent changes may also render such effects visible that would go unnoticed in successive presentation of original and modified pictures. Since visual search mechanisms differ in the manner described above, the oddball paradigm can reveal different aspects of brain processing, such as effects of neural dishabituation. Therefore, introducing the oddball paradigm may be an important methodological addition to the investigation of the change blindness phenomenon.

The early latency of the ERP effect (60-100 ms post-stimulus) suggests that the difference is unlikely to reflect any implicit processing of changes *per se *[28]. Instead, it may be due to preliminary processing of low-level features of images or effects of dishabituation in response to changed features after repetitive identical stimulation. Nevertheless, any such difference indicates that some neural representation exists for the features in which unnoticed changes occur.

The present results cannot be due to the effects of anticipation or task-preparation, which may be the case in a previous study with comparable results from the S1-S2 paradigm, namely, that by Eimer and Mazza ([10]; see also [13]). In their study, Eimer and Mazza also observed a wide-spread positive modulation in brain responses at the early latencies of 30-80 ms and 90-130 ms during change blindness in the S1-S2 paradigm with natural and complex stimuli (groups of faces) with large changes. The authors were unable to interpret this finding simply as a genuine stimulus-related modulation, but instead they proposed that it might be an instance of task-preparation related contingent negative variation, normally elicited by differences of expectations that would be present already before stimulus onset. They conceded that their change blindness trials might have included more trials from sequences with worse task preparation as compared to trials in which participants correctly reported the absence of change, as this could have resulted in the kind of differences in ERPs they observed. In the present study, however, such a bias in change blindness trials is not possible. Namely, because of the pseudo-random presentation of the stimulus types and the fact that data were analyzed only for the standards immediately preceding the deviants (because the occurrence of a standard after the deviant could be expected), both stimulus types were from the same sequence and preceded by numerous identical pictures. Thus, there could have been no systematic difference in subjects' state of preparation, as is possible in the S1-S2 paradigm.

The temporal analyses showed that the onsets and offsets of the differences between responses to (modified) deviant and those to (original) standard images were rather similar at the electrode sites of Fz, Cz, and Pz (significant differences in responses observed between 70 and 104 ms). Unlike these electrode sites, the difference did not quite reach statistical significance by the criterion that we used (p < .01) at the electrode site of Oz. Nonetheless, the offset of the effect seems, on the basis of the temporal analysis and the difference waves, more abrupt at Oz. This may indicate that the responses at this electrode site reflect different brain processes from the ones reflected in responses at the more anterior electrode sites.

In studies of conscious change detection using a S1-S2 or "one-shot" flicker paradigm, a difference in ERP amplitudes at latencies between of 60-150 ms from stimulus onset related to detected stimulus changes in comparison to stimuli containing no change has been observed [10,15,29-32]. However, the polarity of the difference in ERPs varies across studies. In most of the studies [15,29-32], images with detected changes elicited more positive ERPs than those without changes or with undetected changes. In the study of Eimer & Mazza [10], identified changes evoked a negative difference compared to the no-change situation. In the present study, the difference in ERPs to identified changes as compared to no-change images did not reach significance in MANOVA or in the point-by-point temporal analysis, although there was some hint of differential activity at the Oz electrode in the grand average waveforms (Figure 2). The reason why the change related modulation did not reach significance in the present study may be in the differences of psychological states of the subjects. In other studies subjects focused on changes, while in the present study the participants were instructed to ignore the previously identified changes. The results, however, suggest that the modulation of ERPs by unnoticed changes observed in the change blindness trials reflects neural processes that are different from those related to explicit change identification.

One purpose of the study was to make the experimental conditions resemble those of behavioral studies, and therefore participants were allowed to search freely for the change. Moreover, since the main interest and analyses were on the latencies that precede even the most rapid eye-movements evoked by sensory stimulation [33], we decided not to constrain them. We endeavored to avoid any compromising effects of covert visual spatial attention or inhibition of eye-movements on responses to changes that might result from such restrictions. Constraining eye-movements has been suggested to result in obtaining data on active inhibition of eye-movements rather than responses to visual stimulation [34], and it has also been shown to affect change detection performance in the flicker paradigm [35].

## Limitations

Our study is in line with the view that changed features are registered by the neural system during change blindness. However, with the present methods, it is not possible to determine whether the results are due to stimulus novelty, rareness or content of visual change. Also, as we used a conservative criterion of change detection - the changes had to be identified - it is possible that our results do not categorically reflect change blindness and change identification, but also partly some weaker form of change awareness, a phenomenal "sensing" of the changes as reported by Rensink [1]. Also, it is not possible to draw any conclusions of the neural sources of the ERP effects with few electrodes.

## Conclusions

In sum, the present results show that the brain registers visual changes very rapidly, less than 100 ms after the change onset, even when the subjects are not aware of these changes. The results do not support the prevailing view that change detection depends merely on top-down focal attention [1,4]. The data concur with the results of behavioral and neurophysiological studies [5-7,10,12-17] suggesting a role for bottom-up processes in change detection.

## Abbreviations

EEG: Electroencephalogram; ERP: Event-related potentials; vMMN: Visual mismatch negativity; MANOVA: Multivariate analyses of variance.

## Competing interests

The authors declare that they have no competing interests.

## Authors' contributions

PL participated in the conception and design of the study, manufactured the stimuli, carried out electrophysiological measurements, ran the statistical analyses, and drafted the manuscript. JW participated in the conception and design of the study, carried out electrophysiological measurements, preprocessed the electrophysiological data, and helped to interpret the results and to draft the manuscript. PA participated in the conception and design of the study, helped to run the statistical analyses, to interpret the results and to draft the manuscript.
